# Case Report: Hybrid low-grade fibromyxoid sarcoma and sclerosing epithelioid fibrosarcoma of the retroperitoneum

**DOI:** 10.3389/fonc.2026.1673104

**Published:** 2026-02-13

**Authors:** Zhandos Burkitbayev, Aruzhan Zhaksylyk, Baiduisen Ussipbekov, Talgat Uskenbayev, Altay Kerimkulov, Тomiris Sarina, Artem Gogolev, Saltanat Bolsinbekova, Aigerim Sipenova

**Affiliations:** 1 Department of Surgery, National Research Oncology Center, Astana, Kazakhstan; 2 Department of Surgery, School of Medicine, Nazarbayev University, Astana, Kazakhstan

**Keywords:** en-bloc surgical resection, hybrid soft tissue tumor, low-grade fibromyxoid sarcoma, retroperitoneal sarcoma, sclerosing epithelioid fibrosarcoma

## Abstract

Retroperitoneal Sarcomas are rare malignancies that comprise 1%–2% of all malignant tumors. Among them, Low-Grade Fibromyxoid Sarcoma (LGFMS) and Sclerosing Epithelioid Fibrosarcoma (SEF) are ultra-rare sarcomas, especially in retroperitoneum. Hybrid LGFMS/SEF is even rarer, with limited cases reported in literature. We present the case of a 63-years old male with complaints on left hypochondrial discomfort, weight loss, and generalized weakness. Imaging revealed a large retroperitoneal mass with suspicion of local invasion. The patient underwent en-bloc surgical resection, including distal pancreatectomy, splenectomy, and left adrenalectomy. Histopathological examination confirmed a hybrid LGFMS/SEF with characteristic biphasic morphology. Hybrid LGFMS/SEF tumors exhibit features of both LGFMS and SEF sarcoma subtypes. Complete surgical resection remains the primary treatment strategy for localized disease. Because of the rarity of the disease, long-term surveillance is recommended. For advanced diseases, there are limited efficient available treatments necessitating the research of targeted therapies.

## 1 Introduction

Retroperitoneal sarcomas (RS) are rare malignant tumors that account for 1%–2% of all solid malignancies (1). Majority of the sarcoma occur outside the retroperitoneum, and this site comprises only 10-20% of all cases. The overall incidence of RS is estimated to be 0.3–0.4 per 100000 (2).

Low-grade fibromyxoid sarcoma (LGFMS) is a distinct subtype of soft tissue sarcoma characterized by alternating fibrous and myxoid areas and bland spindle cells (3). It most commonly arises in the trunk or extremities and is exceedingly rare in the retroperitoneum. LGFMS predominantly affects young adults, with a slight male predominance (4, 5).

Sclerosing epithelioid fibrosarcoma (SEF) is another rare soft tissue sarcoma, histologically appears as nests of round/oval small epithelioid cells in a dense sclerotic stroma (6). SEF exhibits more aggressive behavior than LGFMS, with local recurrence or distant metastasis—particularly to the lungs and pleura—occurring in approximately 50% of cases (7). Like LGFMS, SEF typically arises in the extremities or trunk (8, 9).

Both LGFMS and SEF are classified as ultra-rare sarcomas. LGFMS occurs with an incidence of 0.18 cases per million (10). SEF’s incidence is less than 1 per million persons, and 100–200 cases were reported in the literature to date (11, 12). LGFMS is associated with more indolent courses, while SEF is associated with more aggressive courses, more recurrence and metastasis rates (13).

Hybrid low-grade fibromyxoid sarcoma/sclerosing epithelioid fibrosarcoma (H-LGFMS/SEF) combines histologic features of both LGFMS and SEF and represents an even rarer entity. In a retrospective analysis by the Ultra-Rare Sarcoma Working Group, 19 cases of localized H-LGFMS/SEF were identified (13), and more recently, the same group reported 23 cases of advanced or metastatic hybrid tumors (14). Due to its rarity, the global incidence of H-LGFMS/SEF remains unknown.

We present a rare case of H-LGFMS/SEF arising in the retroperitoneum of a 63-year-old male. The patient presented with left hypochondrial discomfort, progressive weight loss, and generalized weakness. Physical examination was unremarkable, but imaging revealed a large retroperitoneal mass with possible local invasion. This case is unique due to rare hybrid histology, retroperitoneal location, and successful surgical management without recurrence. The case highlights the clinical presentation, surgical management, histopathological findings, and a brief review of the relevant literature.

## 2 Case presentation

**Figure 1:**
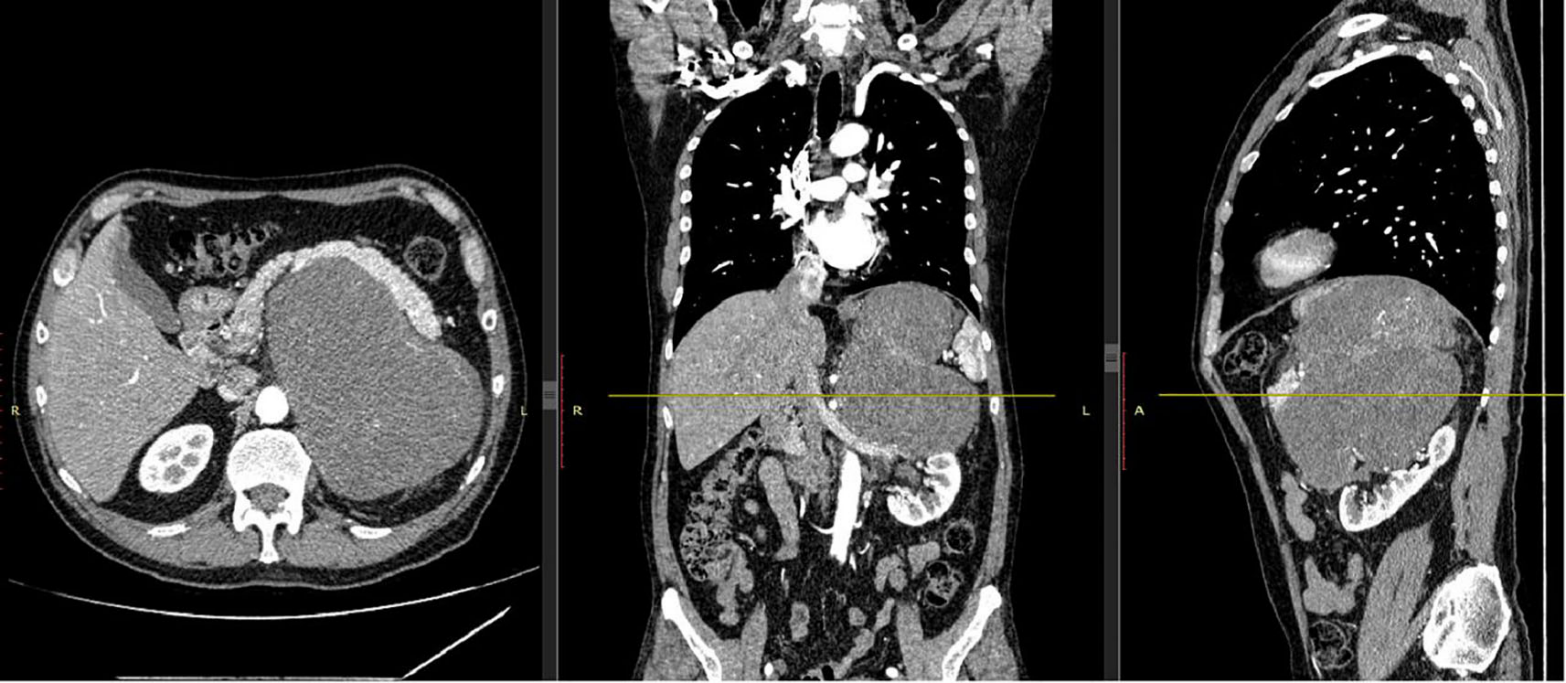
CT imaging of a retroperitoneal mass with mixed density.

A 63-year-old male presented with a two-month history of left upper quadrant discomfort, weight loss (3 kg), and generalized fatigue. Initially evaluated for cardiac causes, his workup ruled out acute coronary syndrome. Abdominal ultrasound and subsequent contrast-enhanced computed tomography (CT) revealed a large retroperitoneal mass measuring 12 × 16.4 × 16.9 cm in the left abdominal cavity, displacing the stomach and spleen and suspected of invading the splenic vein (**Figure 1**). Tumor markers, including CA 19-9, AFP, and CEA, were within normal limits. The patient was referred to an oncology center, where he was diagnosed with retroperitoneal mass based on CT images and recommended for surgical excision of the mass.

On physical examination, the patient is alert, oriented, and able to engage in productive communication appropriately. No cardiovascular or respiratory abnormalities were detected. On abdominal examination, the abdomen is soft and non-tender on palpation. It is asymmetrical due to firm mass measuring 15x12 cm palpated in the left hypochondrium. The patient reports regular bowel movements with normal stool characteristics, and gas passage is unremarkable. Costovertebral tenderness is negative on both sides. The patient urinates independently and urine is of a normal color. Peripheral pulses are palpable, capillary refill is immediate, no peripheral edema.

En-bloc excision of the tumor together with adjacent spleen, distal pancreas, and left adrenal gland was performed. During follow-up, contrast-enhanced CT of the chest and abdomen performed in January 2026 showed no evidence of pulmonary metastases. Metastatic lower paraesophageal lymphadenopathy and findings consistent with gastric carcinoma with regional lymphadenopathy (cT4aN3) were identified. A lesion in the left hepatic lobe requiring further MRI evaluation to differentiate between metastatic disease and hemangioma was also noted.

### 2.1 Timeline

The clinical course and key diagnostic and therapeutic milestones are summarized in **Table 1**.

**Table 1:** Timeline of clinical presentation, diagnostic workup, and treatment.

Date	Clinical event
November 2024	Onset of pain and discomfort. Pain in the left hypochondrium in the supine position, weight loss of 3 kg over a month, general weakness.
January 2025	Ultrasound examination reveals space-occupying formation in the abdominal cavity (in the projection of the tail of the pancreas). Hepatomegaly. Cyst of the right lobe of the liver. Diffuse changes in the liver parenchyma (ecological signs of steatohepatosis). Cholesterosis of the gallbladder (polypoid form). Diffuse changes in the pancreatic parenchyma.
January 2025	CT imaging revealed a space-occupying lesion in the retroperitoneal space on the left without reliable organ affiliation. Tight adjacency, absence of a fat layer between the tumor and the fundus of the stomach, as well as deformation, thread-like caliber of the splenic vein, raise suspicion of invasion. Paraaortic lymphadenopathy at the tumor level. Mediastinal lymphadenopathy. Local pneumofibrosis on the left. Liver cysts. Kidney cysts according to Bosniak 1.
January 2025	Tumor markers from 01/24/2025: CA 19-9 4.04 sd/ml, AFP 2.19 IU/ml, CEA 1.72 ng/ml, diagnosed with “Formation of the retroperitoneal space on the left”.
February 2025	Complete surgical resection performed
February 2025	Macro specimen/biopsy: Retroperitoneal tumor in a single block with the distal part of the pancreas and spleen.

### 2.2 Surgical technique

A midline laparotomy was performed under general anesthesia. Intraoperatively, a large, irregularly shaped retroperitoneal mass was identified displacing the stomach and descending colon to the right and the spleen posteriorly. Intraoperative frozen section biopsy confirmed a malignant mesenchymal tumor, necessitating an en-bloc resection of the involved structures. The tumor involved the superior pole of the spleen and distal pancreas, necessitating splenectomy and distal pancreatectomy. The left adrenal gland was also resected due to proximity. No major vascular involvement was noted. The abdominal cavity was irrigated, two drains were placed, and the incision was closed in layers.

### 2.3 Pathological findings

**Figure 2:**
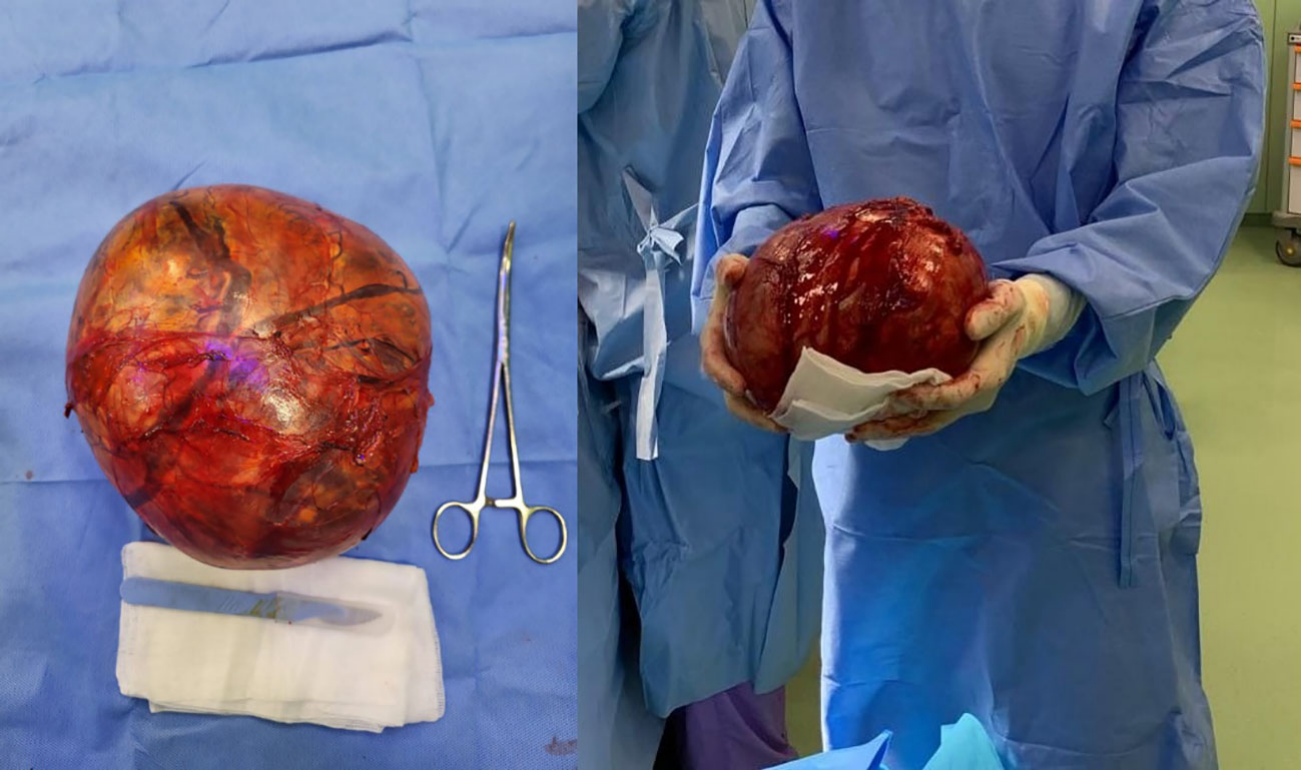
Gross pathology findings of excised retroperitoneal tumor.

Gross Findings: The resected specimen showed a firm, lobulated mass measuring 22x16x10 cm (**Figure 2**).

### 2.4 Microscopic findings

Microscopic examination revealed a biphasic tumor with distinct histological patterns characteristic of both low-grade fibromyxoid sarcoma (LGFMS) and sclerosing epithelioid fibrosarcoma (SEF). One component displayed extensive hyalinized and sclerotic stroma containing sparsely cellular areas composed of small spindle-shaped and epithelioid cells with monomorphic, hyperchromatic nuclei. The second component exhibited a more cellular architecture, featuring richly vascularized myxoid zones with branching capillaries and alternating areas of increased cellularity. Transition between myxoid and sclerotic zones was abrupt and well demarcated.

**Figure 3:**
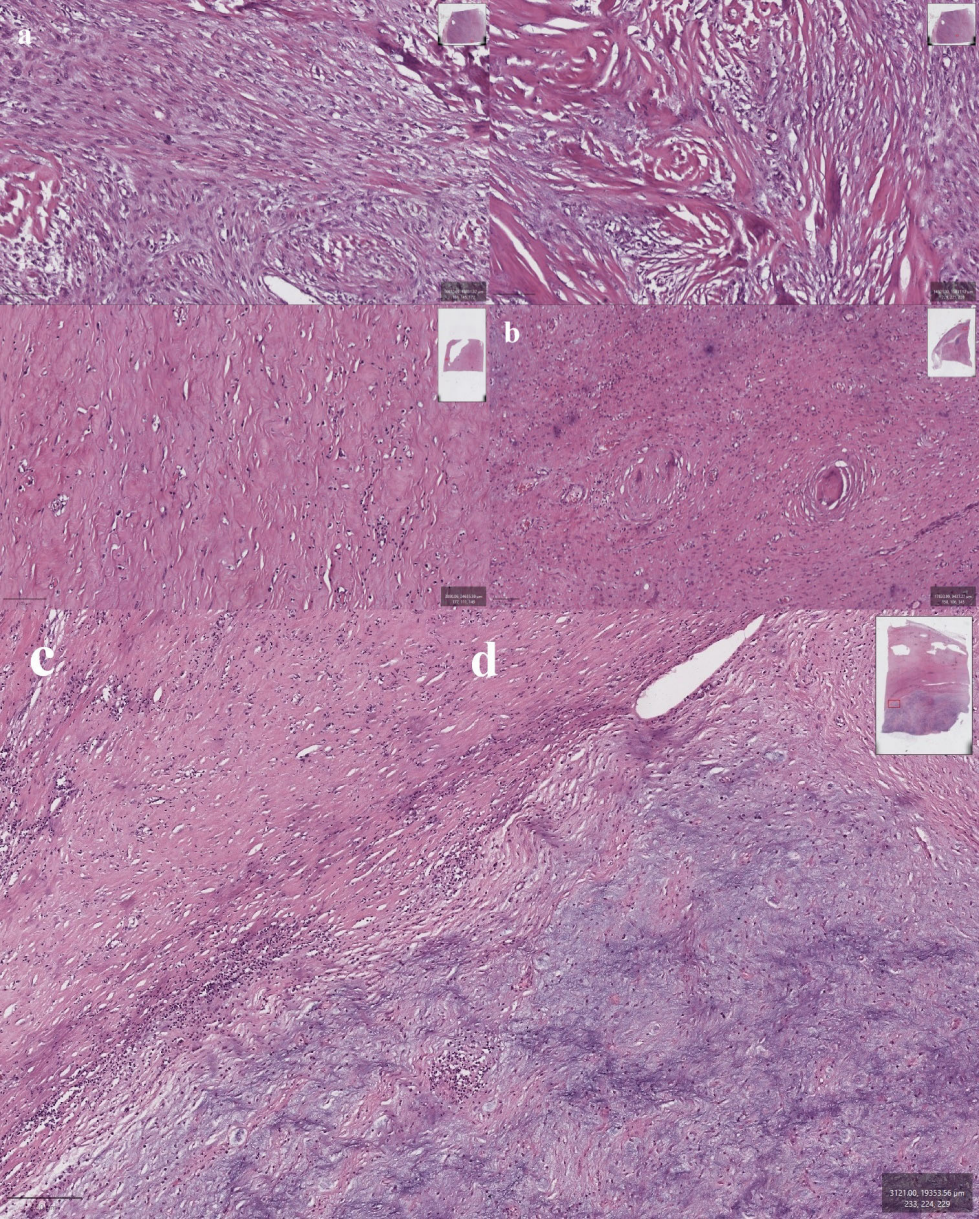
**(a, b)** Sections showing areas with sclerosing epithelioid fibrosarcoma (SEF) morphology (with areas of pronounced sclerosis that contain small spindle-shaped and epithelioid cells); **(c)** bland spindle cells in a myxoid stroma, characteristics of low-grade fibromyxoid sarcoma (LGFMS); **(d)** Collagen rosettes, a distinctive feature of LGFMS; e - Sharp transition between sclerotic and myxoid zones.

Notable features included foci of true metaplastic bone formation, spindle cells arranged in a fascicular growth pattern, and areas of moderate nuclear pleomorphism. In some regions, tumor cells were organized into small clusters and chains. Collagen rosettes—characteristic whorled arrangements of tumor cells around central collagenous cores—were also identified (**Figure 3**).

Based on the combination of these distinct histological features, a final diagnosis of hybrid low-grade fibromyxoid sarcoma and sclerosing epithelioid fibrosarcoma (LGFMS/SEF) of the retroperitoneum was established.

The postoperative course was complicated with fever up to 38°C, increase in WBC, and CRP. Antibiotic therapy was selected and successfully resolved the condition. Two months following the surgical treatment, the patient remains asymptomatic. No adjuvant therapy was initiated, and follow-up surveillance with imaging is planned.

## 3 Discussion

Low-grade fibromyxoid sarcoma and sclerosing epithelioid fibrosarcoma are considered ultra-rare sarcomas. Hybrid LGFMS/SEF tumors, which contain histologic features of both entities, are particularly uncommon and diagnostically challenging. In this report, we present the case of retroperitoneal hybrid LGFMS/SEF in a 63-years old male. The tumor involved adjacent distal pancreas, spleen, and left adrenal gland, necessitating an en-bloc multivisceral resection.

Although image-guided core needle biopsy is generally recommended for retroperitoneal soft tissue sarcomas, biopsy strategies should be individualized and discussed within a multidisciplinary team. In this case, given the radiological impression of a resectable tumor, the heterogeneous appearance of the mass, and concern for sampling error, upfront surgical resection with intraoperative frozen section was considered appropriate.

The retroperitoneal location of this tumor is exceedingly rare, and there are fewer than five previously reported cases in this location (13). A notable feature of the given case is the large size of the tumor measuring 22 × 26 × 10 cm. Retroperitoneal sarcomas often remain asymptomatic until they grow to considerable sizes because of the expansive nature of the retroperitoneal cavity. The patient presented with set of nonspecific symptoms like left hypochondrial discomfort, weight loss, and general weakness, prompting the imaging for large abdominal or retroperitoneal lesions (15).

Review of the literature reveals considerable differences among hybrid LGFMS/SEF tumors. Three notable cases with abdominal involvement include: (1) a 10-year-old girl with renal h-LGFMS/SEF and metastatic disease at diagnosis, carrying an EWSR1-CREB3L1 fusion (16); (2) a 48-year-old male with a small intestinal tumor and liver/bone metastases, showing a rare HEY1-NCOA2 fusion in the liver lesion (17); and (3) a 58-year-old male with a pancreatic tumor and a relatively indolent course, who had an EWSR1 rearrangement on FISH and remained recurrence-free at three months (18). Although SEF is generally associated with a more aggressive invasive and metastatic profile compared to LGFMS, the intimate admixture of both components in this tumor precluded definitive attribution of splenic invasion to a single histological subtype. Our case, similarly to the third case, underwent successful en-bloc resection and has no evidence of recurrence at two months of follow-up.

These cases underscore the genotypic and clinical heterogeneity of hybrid LGFMS/SEF. Although more than 90% of LGFMS and hybrid types carry fusion of FUS-CREB3L fusion, and most SEFs carry EWSR1-CREB3L1 fusions (13, 14, 19), there might be range of atypical genetic rearrangements like HEY1–NCOA2 as mentioned above. The identification of EWSR1 rearrangements may have prognostic implications, as they are more frequently observed in primary SEF and may portray more aggressive behavior (20). Thus, comprehensive molecular profiling plays an essential role in understanding tumor biology and guiding prognosis.

However, molecular testing and MUC4 immunohistochemistry were not feasible in our case, which limits genotypic correlation. Still, histologic features confirmed the biphasic morphology consistent with hybrid LGFMS/SEF. The LGFMS areas showed fibromyxoid stroma with bland spindle cells and rosettes, while the SEF regions contained epithelioid cells in a sclerotic matrix with hyperchromatic nuclei. MUC4 has shown diffuse positivity in hybrid tumors and may help confirm diagnosis in uncertain cases (21).

The mainstay of the treatment for retroperitoneal sarcomas, including, LGFMS and SEF, is complete surgical resection with negative margins (13, 22, 23). Ultra-Rare Sarcoma Working Group’s retrospective study demonstrated that surgical resection in patients with hybrid tumor resulted in no local recurrence. However, nearly half of them developed distant metastases within five years. Pure LGFMS, in contrast, showed excellent long-term outcomes when resected, with near 100% 10-year overall survival in localized disease (13). Neoadjuvant therapy was not administered, as retrospective data from the Ultra-Rare Sarcoma Working Group indicate limited efficacy of systemic treatments in achieving meaningful cytoreduction in hybrid LGFMS/SEF. In addition, systemic therapies and radiotherapy have not demonstrated significant benefit in either localized or metastatic hybrid LGFMS/SEF, underscoring the need for novel therapeutic strategies (13, 14).

Postoperative surveillance is critical, given the potential for delayed metastasis. Current guidelines recommend follow-up every 3–6 months for the first 2–3 years, then every 6 months for the next two years, and annually thereafter (22). Imaging and clinical assessment remain essential in detecting recurrences early. This is especially important given the metastasis rates for H-LGFMS/SEF.

Key Educational Points:

• Diagnostic features: Retroperitoneal sarcomas can grow silently due to their deep location; early imaging is essential when nonspecific symptoms arise.
• Histologic and Molecular Confirmation: Diagnosis of hybrid LGFMS/SEF relies on morphological characteristics. However, immunohistochemistry with MUC4 can help confirming the diagnosis. Molecular profiling can be informative to identify key gene fusions (e.g., FUS–CREB3L2, EWSR1–CREB3L1) and disease course with prognosis.
• Surgical Management: Complete resection with negative margins is critical to optimize local control and survival.
• Surveillance: Close follow-up is required due to the risk of late metastasis and limited efficacy of systemic therapies.

## 4 Conclusion

Hybrid LGFMS/SEF tumors of the retroperitoneum represent a rare and challenging type of tumors. This case illustrates that even large, locally invasive tumors can be successfully resected with favorable short-term outcomes. Nonetheless, the unpredictable metastatic potential of hybrid tumors necessitates long-term follow-up. Molecular profiling, though unavailable in this case, remains essential to inform prognosis and identify novel therapeutic targets. Future studies should aim to better define the biologic spectrum of hybrid LGFMS/SEF and improve outcomes through targeted management strategies.

## Data Availability

The raw data supporting the conclusions of this article will be made available by the authors, without undue reservation.
